# Obesity and pronated foot type may increase the risk of chronic plantar heel pain: a matched case-control study

**DOI:** 10.1186/1471-2474-8-41

**Published:** 2007-05-17

**Authors:** Damien B Irving, Jill L Cook, Mark A Young, Hylton B Menz

**Affiliations:** 1 Musculoskeletal Research Centre, La Trobe University, Bundoora, Victoria 3086, Australia

## Abstract

**Background:**

Chronic plantar heel pain (CPHP) is one of the most common musculoskeletal disorders of the foot, yet its aetiology is poorly understood. The purpose of this study was to examine the association between CPHP and a number of commonly hypothesised causative factors.

**Methods:**

Eighty participants with CPHP (33 males, 47 females, mean age 52.3 years, S.D. 11.7) were matched by age (± 2 years) and sex to 80 control participants (33 males, 47 females, mean age 51.9 years, S.D. 11.8). The two groups were then compared on body mass index (BMI), foot posture as measured by the Foot Posture Index (FPI), ankle dorsiflexion range of motion (ROM) as measured by the Dorsiflexion Lunge Test, occupational lower limb stress using the Occupational Rating Scale and calf endurance using the Standing Heel Rise Test.

**Results:**

Univariate analysis demonstrated that the CPHP group had significantly greater BMI (29.8 ± 5.4 kg/m^2 ^vs. 27.5 ± 4.9 kg/m^2^; *P *< 0.01), a more pronated foot posture (FPI score 2.4 ± 3.3 vs. 1.1 ± 2.3; *P *< 0.01) and greater ankle dorsiflexion ROM (45.1 ± 7.1° vs. 40.5 ± 6.6°; *P *< 0.01) than the control group. No difference was identified between the groups for calf endurance or time spent sitting, standing, walking on uneven ground, squatting, climbing or lifting. Multivariate logistic regression revealed that those with CPHP were more likely to be obese (BMI ≥ 30 kg/m^2^) (OR 2.9, 95% CI 1.4 – 6.1, *P *< 0.01) and to have a pronated foot posture (FPI ≥ 4) (OR 3.7, 95% CI 1.6 – 8.7, *P *< 0.01).

**Conclusion:**

Obesity and pronated foot posture are associated with CPHP and may be risk factors for the development of the condition. Decreased ankle dorsiflexion, calf endurance and occupational lower limb stress may not play a role in CPHP.

## Background

Chronic plantar heel pain (CPHP) is one of the most common conditions affecting the foot and has been reported to account for 15% of all adult foot complaints requiring professional care [1]. It is usually observed in the 40 to 60 year old age bracket, but has been reported in people from 7 to 85 years and appears to be more common in females [2]. Symptoms typically include pain under the medial heel during weight bearing, especially in the morning and at the beginning of weight-bearing activities [1,3].

As with many conditions where the true pathology is unclear, CPHP has become a generalised term encompassing a broad spectrum of conditions affecting the heel, including subcalcaneal bursitis, neuritis, plantar fasciitis and subcalcaneal spur [4,5]. However, plantar fasciitis is considered to be the most common cause of pain and the terms are used interchangeably in the literature [1]. Due to the apparent heterogeneity in the conditions grouped together as CPHP, it is difficult to determine a definitive aetiology for the condition [6].

Many causative factors for CPHP have been hypothesised in the literature and are commonly characterised as intrinsic or extrinsic. Intrinsic factors are characteristics of an individual that predispose them to injury [6]. Those suggested in the literature include limited first metatarsophalangeal joint (MPJ) range of motion (ROM), limited ankle dorsiflexion ROM, leg length discrepancy, reduced heel pad thickness, increased plantar fascia thickness, excessive foot pronation, reduced calf strength, calcaneal spur, older age and increased body mass index (BMI) [1,7,8]. Environmental and circumstantial influences acting upon an individual are known as extrinsic factors, and include prolonged standing, inappropriate shoe fit, previous injury and running surface, speed, frequency and distance per week [1,6,7]. Empirical evidence for most of these factors is limited or absent [9], meaning that the role (if any) of each of these factors in the development of CPHP is poorly understood.

In an attempt to help address this lack of empirical evidence, a matched case-control study was undertaken to examine the association between CPHP and a number of causative factors suggested in the literature. Factors for inclusion into the study were selected because they each had a small amount of evidence supporting an association with CPHP [9], which required further investigation. As it was obviously impractical to examine all factors requiring further investigation, the authors attempted to select those factors that are routinely assessed by clinicians in the management of heel pain. It was hypothesised that pronated foot posture, increased BMI, decreased ankle dorsiflexion ROM, increased occupational lower limb stress and decreased calf endurance would all be associated with CPHP.

## Methods

### Participants

#### Case group

The data source for the case group was a recent CPHP randomised controlled trial [10]. In this trial, advertisements were placed in local and state newspapers requesting volunteers over the age of 18 who had experienced plantar heel pain. Participants with a history of plantar heel tenderness and/or pain upon arising in the morning or on recommencing activity after periods of rest were included in the study. Exclusion criteria included any history of trauma to the heel within the previous 12 weeks, symptoms lasting less than six months, pregnancy, seronegative arthropathies or any skin lesion over the plantar aspect of the heel. Participants who had received a steroid injection or orthotic device or had commenced a conservative treatment such as stretching exercises or heel pads within the previous eight weeks were excluded. Continuation of conservative treatments that had been commenced prior to the eight week period was allowed, however no such participants were identified.

Participants with bilateral heel pain were included and the first 80 eligible participants were used in the study. Case group participants ranged in age from 20 to 82, with a mean of 52 years. Forty-seven participants (59%) were women and the median duration of symptoms was 12 months, ranging from 6 to 96 months.

#### Control group

The control group consisted of 80 participants each individually matched for age (± 2 years) and gender to a case group participant. All participants reported that they had never experienced plantar heel pain. Exclusion criteria were the same as for the case group and control participants were recruited using the same methods as the case group with advertisements placed in newspapers requesting volunteers. The first 80 volunteers that could be matched to a case group participant were included in the study.

### Procedures

Case group data collection was carried out in January 2004 and all testing was undertaken by the same investigator [10]. The same testing equipment and procedures were used to assess the control group over a three-month period from December 2005 to February 2006. All testing of the control group was also all undertaken by the same investigator; however, this was a different investigator to the one that carried out the case group testing. The study was approved by the Faculty of Health Sciences Human Ethics Committee of La Trobe University, and informed consent was obtained from all participants.

### Outcome measures

#### Foot posture

The Foot Posture Index (FPI) [11] was used to assess foot posture. Prior to data collection, both the case and control group investigator were instructed by the same podiatrist with experience in the use of the index.

The FPI is a system for observing and rating static foot posture, incorporating six criteria with the participant standing in a relaxed bipedal position. These criteria include (i) talar head palpation, (ii) observation of curves above and below the lateral malleoli, (iii) frontal plane alignment of the calcaneus, (iv) prominence of the talonavicular joint, (v) congruence of the medial longitudinal arch, and (vi) abduction/adduction of the forefoot on the rearfoot. Each of these criteria are scored on a 5-point scale (ranging from -2 to +2) and the results combined, resulting in a summative score ranging from -12 (highly supinated) to +12 (highly pronated).

The reported inter-tester reliability of the original eight-item FPI has ranged in the literature from an ICC of .62 to .91, while the intra-tester reliability has ranged from .81 to .91 [11]. No reliability statistics have been published for the revised six-item FPI used in this study. The FPI is also a valid measure of foot posture, having been shown to be associated with the midstance position of the foot when walking [11] and to be moderately correlated with arch height measurements taken from x-rays [12].

#### Body mass index

The formula of weight in kilograms divided by the height in meters squared was used to calculate BMI [13]. Participant weight was measured to the nearest tenth of a kilogram using a digital set of scales and height to the nearest centimetre by measuring a point on the wall perpendicular to the superior aspect of the skull.

#### Ankle dorsiflexion range of motion

**Figure 1:**
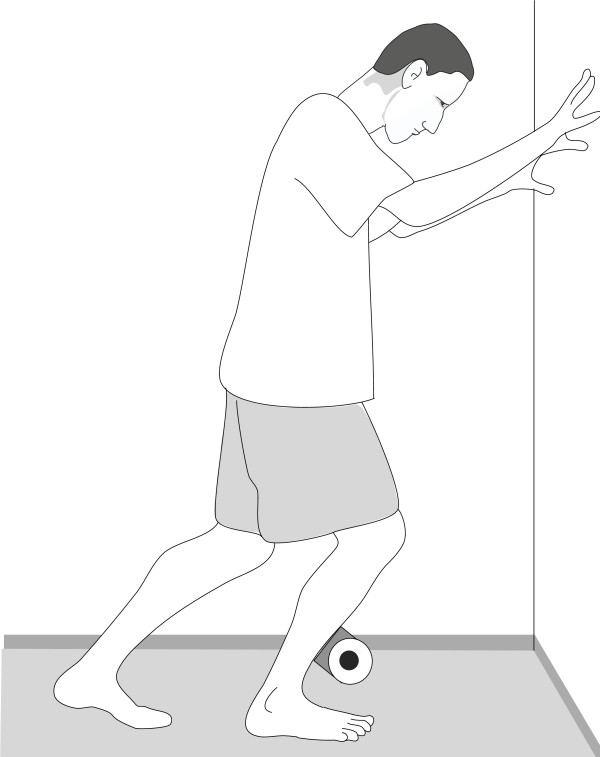
**Dorsiflexion lunge test procedure**. An inclinometer has been placed on the anterior aspect of the tibia. The angle recorded to the vertical was used as a measure of ankle dorsiflexion ROM.

The Dorsiflexion Lunge Test was used to assess ankle dorsiflexion ROM. Testing protocol followed the procedure outlined by Bennell *et al*. [14], which involved the participant lunging their knee as far as possible over their foot without the heel lifting off the floor (Figure 1). At the maximum lunge point, the investigator recorded the angle of the tibia to the vertical (to the nearest tenth of a degree) as a measure of ankle dorsiflexion ROM. Three measures were taken and the mean used for statistical comparisons. This test has been demonstrated to have an intratester reliability of ICC = .98 (SEM = 1.1°) and an intertester reliability of ICC = .99 (SEM = 1.4°) [14].

#### Occupational lower limb stress

The Occupational Rating Scale [15] was used to quantify the amount of stress placed on the lower limb during a typical working day. The scale is a seven-item questionnaire that quantifies time spent sitting, standing/walking, walking on uneven ground, squatting, climbing, lifting/carrying and weight carried. Responses to each question are summed, with a maximum total score of 60 indicating a high level of lower limb stress. The scale has been shown to have excellent test-retest reliability (ICC = .97) [16].

In order to attribute any difference in mean Occupational Rating Scale scores between the case and control groups to the presence of CPHP, co-morbidities were assessed using question 20 of the FHSQ. This question required participants to indicate any conditions for which they were taking medication.

#### Calf endurance

**Figure 2:**
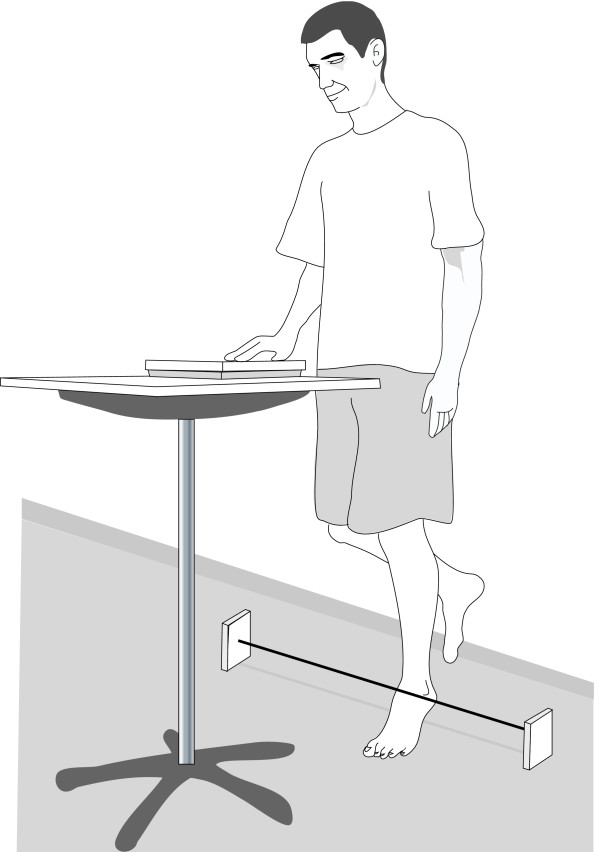
**Standing heel rise test procedure**. The anterior aspect of the ankle is in contact with a string line at maximal ankle plantarflexion. Forward lean is monitored by a set of scales placed on a table. The number of continuous heel raises to touch the sting line was used as a measure of calf endurance.

The Standing Heel Rise Test was used to examine calf muscle performance. Testing protocol followed the procedure outlined by Ross and Fontenot [17], which required the participant to stand on one leg and repeatedly lift the stance limb through a maximum plantar flexion ROM until fatigue (Figure 2). Due to the repetitive nature of the procedure, the test is thought to predominantly assess the endurance capabilities of the calf musculature [17] and therefore the number of heel raises achieved was used as a measure of calf endurance. To ensure that the test was a true indication of calf endurance, participants from the case group were asked to indicate whether heel pain or calf muscle fatigue limited their performance. All participants identified calf muscle fatigue as the limiting factor. The test has been shown to have excellent retest reliability (ICC = .96, SEM = 2.07 repetitions) [17].

### Data analysis

Statistical analyses were conducted using SPSS, version 11.5 for Windows. All variables were explored for normality using the skewness statistic and observations of the normal and de-trended Q-Q plots. With the exception of the Occupational Rating Scale and co-morbidity questionnaire, all variables were compared between the case and control groups using two-tailed independent samples *t*-tests. The Occupational Rating Scale could not be transformed into a normal distribution, so was analysed using the non-parametric Mann-Whitney U test. Chi square tests were used to compare the groups on the prevalence of co-morbidities. Level of significance was set at *P *< 0.01, to account for the fact that multiple comparisons were made between the two groups. For participants with bilateral heel pain, only the more severely affected limb was used in order to meet the independence assumption of statistical analysis [18].

Logistic regression was performed to determine the relative contribution of each of the variables found to differ between the case and control groups in the univariate analyses (i.e. BMI, foot posture and ankle dorsiflexion ROM). Prior to undertaking the logistic regression, FPI scores were dichotomized into pronated (defined as FPI ≥ 4) or not and the Dorsiflexion Lunge Test as excessive (lunge ≥ 47°) or not. These boundaries were selected on the basis of the upper quartile, as no widely accepted cut-off values have been reported in the literature. Body mass index was dichotomized as obese (BMI ≥ 30 kg/m^2^) or not as defined by the National Institutes of Health [13]. Before entering these independent "predictor" variables into the logistic regression, a series of chi-square analyses were undertaken to ascertain whether they were correlated. A non-significant chi-square was calculated in each case and taken as evidence that the predictor variables were not correlated to each other, thereby meeting the independence assumption of logistic regression.

## Results

### Univariate comparisons

When compared to the control group, the case group was found to have a significantly greater mean FPI score (*t *= 2.93, *P *= 0.004), BMI (*t *= 2.85, *P *= 0.005) and mean Dorsiflexion Lunge Test angle (*t *= 4.23, *P *< 0.001). For height and weight analysed individually there was no difference between the groups. The case group had a significantly lower mean Occupational Rating Scale score for mean weight carried (z = -2.98, *P *= 0.003). There was no significant difference between the groups for the prevalence of co-morbidities (Table 1), the Standing Heel Rise Test (Table 2), or for the sitting, standing, uneven ground, squatting, climbing, lifting/carrying or total score sections of the Occupational Rating Scale (Table 2).

### Multivariate comparisons

Results of the logistic regression analysis are shown in Table 3. Of the three variables entered into the model, two were found to be significant independent predictors of CPHP: an FPI ≥ 4 (OR = 3.7, 95% CI 1.6 – 8.7, *P *= 0.002) and a BMI ≥ 30 kg/m^2 ^(OR = 2.9, 95% CI 1.4 – 6.1, *P *= 0.004). The Hosmer and Lemeshow Goodness of Fit Index was non-significant (χ^2 ^= 2.85, *df *= 4, *P *= 0.58), indicating an acceptable goodness of fit. The model classified participants into the CPHP or control group with an accuracy of 66%, indicating that a substantial amount of variance remains unaccounted for.

## Discussion

The purpose of this study was to examine the association between CPHP and a number of commonly hypothesised causative factors. Univariate analyses showed an association between CPHP and increased BMI, pronated foot posture and increased ankle dorsiflexion ROM, whilst occupational lower limb stress and calf endurance showed no association. Multivariate logistic regression showed that those with CPHP were 3.7 times more likely to have a pronated foot posture (FPI ≥ 4) and 2.9 times more likely to be obese (BMI ≥ 30 kg/m^2^).

The association found between a pronated foot type and CPHP is supported by research indicating that increased strain is placed on the plantar fascia when the foot is placed in a pronated position [19-21]. It is important to note that while foot posture has been shown to alter slightly over the course of a lifetime, the change is so slow that it essentially remains constant from one decade to the next [22]. This means that although causality cannot be established in case-control studies, the foot posture of the case group participants is unlikely to have altered after the onset of the condition, and therefore pronated foot posture may also be a risk factor for CPHP.

The association found between increased BMI and CPHP is also supported in the literature, with four of the five previous studies to examine BMI in a non-athletic population also finding an association with increased BMI [9]. The control group appeared to be representative of the wider population, as the proportion of the group found to be obese (BMI ≥ 30 kg/m^2^) was the same value as for the Australian population in the 45–54 year age bracket (21%) [23]. Due to the fact that this study cannot establish causality, it is unclear whether increased BMI existed in the case group participants prior to the development of CPHP, or whether the pain associated with the condition caused participants to reduce their physical activity, thereby leading to an increase in BMI. However, it is plausible that increased BMI may be a risk factor for CPHP, as individuals with increased BMI experience higher vertical forces under the heel during gait [24], leading to higher internal stresses within the heel [25], which may lead to damage of soft tissue structures and the development of symptoms.

The identified association between increased ankle dorsiflexion ROM and CPHP was contrary to the common clinical perspective that decreased ankle dorsiflexion ROM is a causative factor for CPHP. This hypothesis is based on the theory that equinus (ankle dorsiflexion less than 10°) during gait causes abnormal compensatory pronation of the subtalar joint, which in turn increases stress on the plantar fascia [26]. Although this theory is widely accepted, research evidence to support it is weak [27]. Cornwall and McPoil [27] found that a mild-to-moderate loss of passive dorsiflexion ROM (the study group had a mean ROM of 9.6°) had little or no effect on the frontal plane function of the rearfoot during the stance phase of gait. The participants were found to compensate for their dorsiflexion deficit with alterations in gait timing. This may explain why only one of three previous case-control studies found an association between decreased ankle dorsiflexion ROM and CPHP [9].

A possible explanation for the identified association with increased ankle dorsiflexion ROM is that a non-linear relationship may exist between ankle dorsiflexion ROM and plantar fascia strain. If the relationship were U-shaped, both extremes of movement (increased and decreased ROM) would predispose to CPHP. To further substantiate this hypothesis, research would be required to determine whether increased translation of the tibia over the foot increases strain on the plantar fascia.

A criticism of the Dorsiflexion Lunge Test is that the test procedure makes no effort to control for pronation or supination of the foot. As increased subtalar joint pronation is known to increase the amount of dorsiflexion that can occur at the midtarsal joint, it is plausible that the increased ankle dorsiflexion ROM observed in the case group may have been due to the fact that the group was also found to have a more pronated foot posture [28]. However, chi-square analysis indicated that scores for the Dorsiflexion Lunge Test and FPI were not correlated (data not shown), indicating that dorsiflexion was likely to be increased independent of foot posture in the case group participants.

A number of differences exist between the current study and previous literature with regard to dorsiflexion testing methods and results. All previous studies to address the association between ankle dorsiflexion and CPHP examined subjects with the knee extended [9]. Since knee extension biases the gastrocnemius muscle whilst knee flexion has a Soleus bias, it is possible that tightness in the gastrocnemius muscle may have gone undetected in the case group. Also, the Dorsiflexion Lunge Test scores reported in the current study are slightly lower than those documented in the literature [14,29]. Because ankle dorsiflexion ROM decreases with age [30], this difference is likely to be due to the fact that the mean participant age in the current study was approximately 30 years greater than in these previous studies. In summary, the findings of the current study question the role of decreased ankle dorsiflexion ROM in the development of CPHP and suggest increased ankle dorsiflexion ROM as a previously unconsidered causative factor.

Prolonged standing is often cited as a causative factor for CPHP [1,6,7], based on the theory that prolonged tensile loading of the plantar fascia predisposes individuals to the condition [31]. There is a weak level of evidence to support an association between prolonged standing and CPHP [9], however, no previous study has adequately defined prolonged standing. Consequently, there are no data to indicate what activities are commonly performed whilst standing and therefore the nature of the stresses placed on the lower limb [9]. This study was the first to examine prolonged standing in detail, using the Occupational Rating Scale to quantify the stresses placed on the lower limb during an average working day.

As there was no significant difference in the presence co-morbidities between the case and control groups (Table 1), any differences observed between the groups on the Occupational Rating Scale can be cautiously attributed to CPHP. However, no association was found between CPHP and average time spent sitting, standing, walking on uneven ground, squatting, climbing, lifting/carrying or total stress placed on the lower limb. The one significant difference found between the groups indicated an association between CPHP and reduced weight carried. Although this association was identified, it is unlikely that this factor has a role in the development of CPHP. It is more likely that the pain associated with CPHP causes sufferers to carry less weight than they otherwise would.

Although no association was identified with CPHP, it is unclear from these results whether occupational lower limb stress plays a role in the development of the condition. Due to the case-control design of the current study, it is possible that the case group participants experienced higher occupational lower limb stress prior to developing CPHP. The participants may have simply reduced their activity levels, as a consequence of their pain, to a level comparable to the control group. As participants were asked to answer the Occupational Rating Scale according to their current work status, no retrospective comments can be made regarding the association between CPHP and past working history. However, keeping this limitation in mind, it can be cautiously speculated that greater occupational lower limb stress may not be a risk factor for CPHP as previously thought.

No association was identified between calf endurance and CPHP. The Standing Heel Rise Test scores reported for the case and control groups were substantially lower than those reported in the literature, however, this was to be expected. Ross and Fontenot [17] examined a far younger (21.2 ± 1.3 years) and more physically active sample of air force cadets and Lunsford and Perry [32] examined a younger (male: 34.7 ± 8.5; female: 29.3 ± 5.0) sample using a test procedure that allowed a reduction of up to 50% in plantarflexion ROM before termination. As with occupational lower limb stress, it can be speculated from these findings that decreased calf endurance may not play a role in the development of CPHP.

The findings of this study need to be interpreted in light of a number of study limitations. A different investigator was used for each group and no pilot study was conducted to examine the correlation between the investigators for any of the outcome measures used. The Dorsiflexion Lunge Test and Standing Heel Rise Test have structured protocols and the Dorsiflexion Lunge Test has demonstrated high inter-tester reliability in previous studies [14]. It is therefore unlikely that a change in investigator would have substantially altered the results of these measures. The FPI protocol involves a degree of subjectivity due to its observational nature and a change in examiner may have influenced the results. However, the authors are confident that the procedure is reliable enough between examiners to dichotomise participants as having pronated feet or not. Finally, as previously acknowledged, a case-control study cannot imply causation. As such, further research is required to definitively establish whether the associated factors identified in this paper are in fact risk factors for CPHP.

A representative clinical population with heel pain was used in this study; participants were included according to clinical signs and symptoms (chronic plantar heel pain) rather than diagnostic imaging. As CPHP can include a range of pathologies affecting the heel including plantar fasciitis, sub calcaneal bursitis, calcaneal periostitis and subcalcaneal spur, a pain diagnosis was considered the most appropriate method of selecting participants. These conditions can exhibit a combination of osseous and soft tissue pathologies (including calcaneal spurs, plantar fascial thickening, cortical irregularities and fat pad abnormalities [4]) that have variable imaging findings [33-35]. The sample would have been reduced to a specific sub-group of people with CPHP if a single imaging modality had been used as the inclusion criteria. Furthermore, diagnostic imaging is not always necessary for the diagnosis of CPHP, and many health professionals who frequently treat the condition (such as podiatrists and physiotherapists) rely on clinical criteria. We therefore believe that the use of a clinical diagnosis for inclusion into the CPHP group provides results that can be generalised to the broader population of people seeking treatment for heel pain.

A final limitation is that the overall classification accuracy of the model was relatively low (66% of cases correctly classified), which indicates that there may be other variables of importance that were not included in our test battery. Further research is required to determine whether the inclusion of other postulated risk factors can improve the classification accuracy of the multivariate model.

## Conclusion

Obesity and pronated foot posture are associated with CPHP and may be risk factors for the development of the condition. Decreased ankle dorsiflexion, decreased calf endurance and occupational lower limb stress do not appear to play a role in CPHP.

## Competing interests

The author(s) declare that they have no competing interests.

## Authors' contributions

DBI participated in the design of the study, was involved in data collection and prepared the manuscript. JLC conceived of the study, participated in its design and coordination and helped to draft the manuscript. MAY was involved in data collection and drafting of the manuscript and HBM participated in the design of the study, performed the statistical analysis and helped to draft the manuscript. All authors read and approved the final manuscript.

## Pre-publication history

The pre-publication history for this paper can be accessed here:



## Figures and Tables

**Table 1:** Prevalence of co-morbidities in the case and control groups

Co-morbidity	Case group	Control group
Diabetes	4 (5)	3 (4)
Osteoarthritis	4 (5)	6 (8)
High blood pressure	15 (19)	15 (19)
Heart disease	6 (8)	6 (8)
Lung disease	7 (9)	7 (9)
Hormone replacement therapy	4 (5)	7 (9)
High cholesterol	11 (14)	8 (10)
Thyroid disease	6 (8)	3 (4)

Data are *n *(%). Co-morbidities were defined as any self-reported condition for which a participant was currently taking medication. Chi square analysis showed no significant difference between the groups for any co-morbidity.

**Table 2:** Case and control group results for all univariate comparisons

Variable	Case group	Control group	*P *value
Height (m)^1^	1.69 (0.09)	1.69 (0.08)	.504
Weight (kg)^1^	84.8 (17.4)	79.0 (16.0)	.029
Body mass index (kg/m^2^)^1^	29.8 (5.4)	27.5 (4.9)	.005
Foot posture index^1^	2.4 (3.3)	1.1 (2.3)	.004
Standing heel rise test (repetitions)^1^	17.3 (9.7)	14.4 (8.7)	.050
Dorsiflexion lunge test (°)^1^	45.1 (7.1)	40.5 (6.6)	<.001
Occupational rating scale^2^			
Sitting	6 (2)	4 (4)	.578
Standing	6 (4)	6 (4)	.907
Uneven ground	1 (2)	2 (2)	.092
Squatting	1 (2)	1 (2)	.028
Climbing	2 (4)	0 (2)	.558
Lifting or carrying	2 (2)	2 (2)	.236
Weight carried	1 (2)	2 (1)	.003
Total score	17 (12)	22 (11)	.108

^1 ^Data are mean (S.D.), and differences between groups were analysed using independent samples *t*-tests.

^2 ^Data are median (interquartile range), and differences between the groups were analysed using Mann Whitney U-tests. Occupational rating scale subscales can range from 0 (lowest level of lower limb stress) to 10 (highest level of lower limb stress).

**Table 3:** Case and control results for all multivariate comparisons

	No of Cases (*n *= 80)	No of Controls (*n *= 80)	β weight	SE	Wald	Odds ratio (95% CI)	*P *value
Pronated (FPI 4 – 10)	26 (33%)	10 (13%)	1.31	0.43	9.21	3.7 (1.6 – 8.7)	.002
Excessive dorsiflexion (DLT 47.0 – 69.8°)	26 (33%)	15 (19%)	0.69	0.40	2.92	2.0 (0.9 – 4.4)	.088
Obese (BMI 30.0 – 46.3 kg/m^2^)	31 (39%)	17 (21%)	1.08	0.38	8.26	2.9 (1.4 – 6.1)	.004
Constant	-	-	-0.77	0.25	9.83	0.46	.002

SE, standard error; CI, confidence interval; FPI, foot posture index; DLT, dorsiflexion lunge test; BMI, body mass index.

## References

[B1] Rome K (2005). Heel pain: diagnosis and management. Podiatry Now.

[B2] Ozdemir H, Yilmaz E, Murat A, Karakurt L, Kursad-Poyraz A, Ogur E (2005). Sonographic evaluation of plantar fasciitis and relation to body mass index. European Journal of Radiology.

[B3] Hunt GC, Sneed T, Hamann H, Chisam S (2004). Biomechanical and histiological considerations for development of plantar fasciitis and evaluation of arch taping as a treatment option to control associated plantar heel pain: A single-subject design. The Foot.

[B4] Narvaez JA, Narvaez J, Ortega R, Aguilera C, Sanchez A, Andia E (2000). Painful heel: MR imaging findings. Radiographics.

[B5] Rome K (1997). Anthropometric and biomechanical risk factors in the development of plantar heel pain – a review of the literature. Physical Therapy Reviews.

[B6] Rome K, Howe T, Haslock I (2001). Risk factors associated with the development of plantar heel pain in athletes. The Foot.

[B7] Buchbinder R (2004). Plantar fasciitis. New England Journal of Medicine.

[B8] Allen R, Gross M (2003). Toe flexors strength and passive extension range of motion of the first metatarsophalangeal joint in individuals with plantar fasciitis. Journal of Orthopaedic and Sports Physical Therapy.

[B9] Irving DB, Cook JL, Menz HB (2006). Factors associated with chronic plantar heel pain: A systematic review. Journal of Science and Medicine in Sport.

[B10] Young MA, Cook JL, Webster KE (2006). The effect of topical wheatgrass cream on chronic plantar fasciitis: A randomised, double blind, placebo controlled trial. Complementary Therapies in Medicine.

[B11] Redmond AC, Crosbie J, Ouvrier RA (2006). Development and validation of a novel rating system for scoring standing foot posture: The Foot Posture Index. Clinical Biomechanics.

[B12] Menz HB, Munteanu SE (2005). Validity of 3 clinical techniques for the measurement of static foot posture in older people. Journal of Orthopaedic and Sports Physical Therapy.

[B13] National Institutes of Health (1998). Clinical guidelines on the identification, evaluation, and treatment of overweight and obesity in adults: the evidence report.

[B14] Bennell KL, Talbot RC, Wajswelner H, Techovanich W, Kelly DH, Hall AJ (1998). Intra-rater and inter-rater reliability of a weight-bearing lunge measure of ankle dorsiflexion. Australian Journal of Physiotherapy.

[B15] Noyes FR, Mooar LA, Barber SD (1991). The assessment of work-related activities and limitations in knee disorders. The American Journal of Sports Medicine.

[B16] Barber-Westin SD, Noyes FR, McCloskey JW (1999). Rigorous statistical reliability, validity, and responsiveness testing of the Cincinnati Knee Rating System in 350 subjects with uninjured, injured, or anterior cruciate ligament-reconstructed knees. The American Journal of Sports Medicine.

[B17] Ross MD, Fontenot EG (2000). Test-retest reliability of the standing heel-rise test. Journal of Sports Rehabilitation.

[B18] Menz HB (2004). Two feet, or one person? Problems associated with statistical analysis of paired data in foot and ankle medicine. The Foot.

[B19] Kwong PK, Kay D, Voner RT (1988). Plantar fasciitis: mechanics and pathomechanics of treatment. Clinical Sports Medicine.

[B20] Sarrafian SK (1987). Functional characteristics of the foot and plantar aponeurosis under tibiotalar loading. Foot & Ankle International.

[B21] Wright DG, Rennels DC (1964). A study of the elastic properties of the plantar fascia. Journal of Bone and Joint Surgery.

[B22] Staheli LT, Chew DE, Corbett M (1987). The longitudinal arch. A survey of eight hundred and eighty-two feet in normal children and adults. The Journal of Bone and Joint Surgery: American Volume.

[B23] Australian Bureau of Statistics 2004 – 05 National health survey: Summary of results (No. 4364.0).

[B24] Hills AP, Hennig EM, McDonald M, Bar-Or O (2001). Plantar pressure differences between obese and non-obese adults: a biomechanical analysis. International Journal of Obesity and Related Metabolic Disorders.

[B25] Spears IR, Miller-Young JE, Waters M, Rome K (2005). The effect of loading conditions on stress in the barefooted heel pad. Medicine and Science in Sports and Exercise.

[B26] Hill RS (1995). Ankle equinus: Prevalence and linkage to common foot pathology. Journal of the American Podiatric Medical Association.

[B27] Cornwall MW, McPoil TG (1999). Effect of ankle dorsiflexion range of motion on rearfoot motion during walking. Journal of the American Podiatric Medical Association.

[B28] Burns J, Crosbie J (2005). Weight bearing ankle dorsiflexion range of motion in idiopathic pes cavus compared to normal and pes cavus feet. The Foot.

[B29] Malliaras P, Cook JL, Kent P (2006). Reduced ankle dorsiflexion range may increase the risk of patellar tendon injury among volleyball players. Journal of Science and Medicine in Sport.

[B30] Scott G, Menz HB, Newcombe L Age-related differences in foot structure and function. Gait and Posture.

[B31] Riddle DL, Pulisic M, Pidcoe P, Johnson RE (2003). Risk factors for plantar fasciitis: A matched case-control study. Journal of Bone and Joint Surgery.

[B32] Lunsford BR, Perry J (1995). The standing heel rise test for ankle plantar flexion: criterion for normal. Physical Therapy.

[B33] Berkowitz JF, Kier R, Rudicel S (1991). Plantar fasciitis: MR imaging. Radiology.

[B34] Osborne HR, Breidahl WH, Allison GT (2006). Critical differences in lateral X-rays with and without a diagnosis of plantar fasciitis. Journal of Science and Medicine in Sport.

[B35] Cardinal E, Chhem RK, Beauregard CG, Aubin B, Pelletier M (1996). Plantar fasciitis: sonographic evaluation. Radiology.

